# Risk factors for postoperative fever after laparoscopic adrenalectomy focusing on hormones produced: a case control study

**DOI:** 10.1186/s12894-024-01469-w

**Published:** 2024-04-18

**Authors:** Mizuki Izawa, Toshikazu Takeda, Tadatsugu Anno, Tomohiro Iwasawa, Yota Yasumizu, Nobuyuki Tanaka, Kazuhiro Matsumoto, Shinya Morita, Takeo Kosaka, Ryuichi Mizuno, Hiroshi Asanuma, Mototsugu Oya

**Affiliations:** Department of Urology, Keio University School of Medicine, 35 Shinanomachi, Shinjuku-Ku, Tokyo, 160-8582 Japan

**Keywords:** Laparoscopic adrenalectomy, Postoperative fever, Pheochromocytoma, Cushing syndrome, Hormone-producing tumors, Postoperative complications

## Abstract

**Background:**

Laparoscopic adrenalectomy is widely performed for a number of hormone-producing tumors and postoperative management depends on the hormones produced. In the present study, we conducted a retrospective analysis to clarify the risk factors for postoperative complications, particularly postoperative fever after laparoscopic adrenalectomy.

**Methods:**

We analyzed 406 patients who underwent laparoscopic adrenalectomy at our hospital between 2003 and 2019. Postoperative fever was defined as a fever of 38 °C or higher within 72 h after surgery. We investigated the risk factors for postoperative fever after laparoscopic adrenalectomy.

**Results:**

There were 188 males (46%) and 218 females (54%) with a median age of 52 years. Among these patients, tumor pathologies included 188 primary aldosteronism (46%), 75 Cushing syndrome (18%), and 80 pheochromocytoma (20%). Postoperative fever developed in 124 of all patients (31%), 30% of those with primary aldosteronism, 53% of those with pheochromocytoma, and 8% of those with Cushing syndrome. A multivariate logistic regression analysis identified pheochromocytoma and non-Cushing syndrome as independent predictors of postoperative fever. Postoperative fever was observed in 42 out of 80 cases of pheochromocytoma (53%), which was significantly higher than in cases of non-pheochromocytoma (82/326, 25%, *p* < 0.01). In contrast, postoperative fever developed in 6 out of 75 cases of Cushing syndrome (8%), which was significantly lower than in cases of non-Cushing syndrome (118/331, 35.6%, *p* < 0.01).

**Conclusion:**

Since postoperative fever after laparoscopic adrenalectomy is markedly affected by the hormone produced by pheochromocytoma and Cushing syndrome, it is important to carefully consider the need for treatment.

## Background

Adrenal tumors include many hormonally active tumors. A previous study reported that 69–75% of adrenal tumors are nonfunctional: 10–12% are Cushing syndrome, 7–10% pheochromocytoma, 2.5–6% primary aldosteronism (PA), 8–11% adrenocortical carcinoma, and 5–7% metastatic from other cancers [1]. The surgical resection of adrenal tumors is preferable if they are accompanied by hormone production and are symptomatic, and laparoscopic surgery has recently become the golden standard [2]. However, it differs from surgery for other diseases in that the condition of a patient in the perioperative period may markedly change due to fluctuations in serum hormone levels. In endocrine diseases, surgery may induce the secretion of febrile substances, such as cytokines and interleukins [3], and postoperative fever is an important vital sign that may detect changes in a patient’s condition at an early stage. Difficulties are associated with establishing whether postoperative fever is due to a fluctuation in blood hormone levels or is an indicator of infection. Most surgeons agree that there are many cases of transient fever immediately after surgery that are clinically uneventful and recover without therapeutic interventions [4, 5]. Although postoperative fever after major surgical procedures is a relatively common event that occurs in between 10 and 40% of patients [6], limited information is currently available on the incidence of fever after adrenal surgery. The overall incidence of complications associated with laparoscopic adrenalectomy was previously reported to be 9.5% (range 2.9–20), with the most common postoperative complication being bleeding (21.5% of all cases), followed by wound complications (13%). The exact incidence of postoperative fever has not yet been reported, while other complications, including fever, occur at an incidence of approximately 9.5% and include thromboembolism, neurological abnormalities, anemia, neutropenia, nausea, headache, shoulder tip pain, and subcutaneous and mediastinal emphysema [2]. In the present study, we focused on postoperative complications and aimed to clarify the risk factors for postoperative fever after laparoscopic adrenalectomy.

## Methods

After approval by the Institutional Review Board, we analyzed 434 patients who underwent surgery for adrenal tumors at our hospital between December 2003 and April 2019. Twenty-eight patients who underwent open surgery and 3 who underwent laparoscopic adrenalectomy using a retroperitoneal approach, and remaining 403 patients were examined. Early postoperative fever was defined as a fever of 38 °C (100.4℉) or higher within 72 h after surgery [7]. These 406 patients were divided into two groups: a fever group with body temperature > 38 °C within 72 h after surgery, and a non-fever group. The median postoperative hospital stay in the present study was 6 days (range, 3–23). We retrospectively collected the following data from each patient: age, sex, body mass index, the American Society of Anesthesiologists score, previous abdominal surgery, comorbidities, type of disease, tumor localization, tumor size, pneumoperitoneum time, estimated blood loss, and preoperative blood data.

Preoperative blood data were checked as an index to evaluate susceptibility to infection (white blood cells, total protein, albumin, and C-reactive protein). In cases of pheochromocytoma, 24-h urinary catecholamine levels, such as epinephrine (E), norepinephrine (NA), dopamine (D), metanephrine (MN), normetanephrine (NMN), and vanillylmandelic acid (VMA), measured prior to the histological confirmation of the tumor pathology of pheochromocytoma were tabulated for each patient. Systolic blood pressure and heart rate during surgery were measured. In cases of Cushing syndrome, preoperative serum glucocorticoid and adrenocorticotrophic hormone (ACTH) levels were also examined. Continuous variables in the table are denoted by mean ± SD.

SPSS ver.25 statistical software package (IBM Corp., Armonk, NY, USA) was used for each analysis, with the χ2 test for nominal variables and the Mann–Whitney test for continuous variables. Results were interpreted as significant at a *p* value < 0.05.

## Results

Table 1 shows the background of 406 patients who underwent laparoscopic adrenalectomy. A total of 263 of these patients (64%) (188 with PA and 75 with Cushing syndrome) had benign tumors with endocrine activity, 80 (20%) had pheochromocytoma, and 63 (16%) had non-functioning adrenal adenoma, adrenal carcinoma, metastatic tumors, cystic tumors, myelolipoma, neurinoma, ganglioneuroma, hemangioma, fibrosis, lymphangioma, teratoma, pigmented nodular adrenocortical disease, perivascular epithelioid cell tumor, or hematoma. The median age of patients at the time of surgery was 52 years (range, 8–90). The median pneumoperitoneum time was 75 min (range, 26–504). Median estimated blood loss was 30.0 mL, and only one patient required a blood transfusion. Postoperative complications were observed in 14 cases (3.4%): 5 cases of postoperative infection, 2 of retroperitoneal hematoma, 2 of cardiovascular events, 1 of cerebral infarction, 1 of severe hypoglycemia, 1 of renal infarction, 1 of umbilical hernia, and 1 of chylorrhea.

**Table 1:** Patient characteristics in non-fever and fever group

	Non-fever group	Fever group	*p* value
**Number of patients**	282(69%)	124(31%)	
**Gender**			0.022*
Male	120(43%)	68(55%)	
Female	162(57%)	56(45%)	
**Age(years)**	52.2 ± 12.5	51.5 ± 13.4	0.314
**Tumor localization**			0.258
Right	102(36%)	47(38%)	
Left	174(62%)	77(62%)	
Bilateral	6(2%)	0(0%)	
**Previous abdominal surgery**			0.550
Yes	62(21%)	24(19%)	
No	220(79%)	100(81%)	
**Diabetes**			0.033*
Yes	20(7%)	17(14%)	
No	262(93%)	107(86%)	
**ASA score**			0.467
< 3	152(97%)	81(94%)	
≧3	9(3%)	7(6%)	
**BMI**	23.01 ± 3.68	22.98 ± 3.71	0.444
**Pneumoperitoneum time (min.)**	71 ± 46.0	86 ± 52.6	0.005*
**Blood loss(ml)**	75 ± 47.9	75 ± 47.9	0.061
**Tumor size(mm)**	25 ± 17.6	24 ± 22.3	0.262

*ASA score* American Society of Anesthesiologists, *BMI* body mass index ^*^p＜0.05

A total of 124 out of 406 patients (31%) developed postoperative fever, the median duration of which was 1.4 days (range, 1–4). More than 50% of patients recovered within one day and were in a good general condition. Among the 124 patients in the fever group, one had fever due to surgical site infection with pheochromocytoma and the other due to pleurisy with PA. The percentage of male patients was significantly higher in the fever group (55% vs. 45%, *p* = 0.022). The percentage of diabetes mellitus patients was also significantly higher in the fever group (14% vs. 7%, *p* = 0.033). The median pneumoperitoneum time (86.0 min vs 71.0 min, *p* = 0.006) were significantly longer in the fever group.

The pathological features of the entire population are shown in Table 2. Postoperative fever was observed in 42 out of the 80 cases of pheochromocytoma, (52%), which was significantly higher than in cases of non-pheochromocytoma (82/326, 25%, *p* < 0.01). In contrast, postoperative fever developed in only 6 out of the 75 cases of Cushing syndrome, (8%), which was significantly lower than in cases of non-Cushing syndrome (118/331, 35.6%, *p* < 0.01). Three patients (Cushing, PA, and non-functioning adenoma) underwent laparoscopic adrenalectomy by retroperitoneal approach. Patients with Cushing and PA experienced postoperative fever.

**Table 2:** Pathological summary of study patients

			Fever		
		Total	Non-fever group	Fever group	*p* value
		*n* = 406	*n* = 282	*n* = 124	
**Primary aldosteronism**	*Yes*	188(46%)	132(47%)	56(46%)	0.422
	*No*	218(53%)	150(53%)	68(54%)	
**Pheochromocytoma**	*Yes*	80(20%)	38(14%)	42(34%)	< 0.001^*^
	*No*	326(80%)	244(86%)	82(66%)	
**Cushing syndrome**	*Yes*	75(18%)	69(25%)	6(5%)	< 0.001^*^
	*No*	331(82%)	213(75%)	118(95%)	
**Others**^***a***^	*Yes*	63(16%)	43(16%)	20(17%)	0.464
	*No*	343(84%)	239(84%)	104(83%)	

^a^Other pathologies include: Cortical adenoma/adenoma (*n* = 14), metastasis (*n* = 10), adrenal cyst/pseudocyst (*n* = 9), myelolipoma (*n* = 7), neurinoma (*n* = 7), ganglioneuroma (*n* = 5), adrenal carcinoma (*n* = 3), hemangioma (*n* = 1), fibrosis (*n* = 1), lymphangioma (*n* = 1), teratoma (*n* = 1), pigmented nodular adrenocortical disease (*n* = 1), perivascular epithelioid cell tumor (*n* = 1), and hematoma (*n* = 1) ^*^p＜0.05

The multivariate analysis identified pheochromocytoma (*p* = 0.024, odds ratio 2.211, 95% confidence interval (CI) 1.110–4.445), non-Cushing syndrome (*p* < 0.001, odds ratio 0.171, 95% CI 0.063–0.465), and diabetes (*p* = 0.032, odds ratio 2.250, 95% CI 1.070–4.728) as independent predictors of postoperative fever (Table 3).

**Table 3:** Univariate and multivariate analyses of factors affecting postoperative fever

		Univariate	Multivariate		
	Categories	*p* value	OR	95%CI	*p* value
**Gender**	Male vs. Female	0.022*			
**Age(years)**	< 52 vs. ≥ 52	0.553			
**Tumor localization**	Right vs. Left vs. Bilateral	0.258			
**Tumor pathology**		< 0.001*			
	Others	Reference			Reference
	Cushing syndrome	< 0.001*	0.171	(0.063–0.465)	< 0.001^*^
	Pheochromocytoma	< 0.001*	2.221	(1.110–4.445)	0.024^*^
**Previous abdominal surgery**	Yes vs. No	0.550			
**Diabetes**	Yes vs. No	0.033*	2.250	(1.070–4.728)	0.032^*^
**ASA score**	< 3 vs. ≥ 3	0.467			
**BMI**	< 23 vs. ≥ 23	0.948			
**Pneumoperitonium time(min.)**	< 75 vs. ≥ 75	0.006*			
**Blood loss(ml)**	< 30 vs. ≥ 30	0.183			
**Tumor size(mm)**	< 24 vs. ≥ 24	0.976			

*ASA score* American Society of Anesthesiologists, *BMI* body mass index, *OR* odds ratio, *CI* confidence interval ^*^p＜0.05

Among 80 patients with pheochromocytoma, 24-h urinary data were available for 33 cases. No significant differences were observed in urinary E, NA, D, MN, NMN, or VMA levels between the fever and non-fever groups. Data on oral medications were available for 50 of the 80 pheochromocytoma cases, with doxazosin as an alpha blocker; the preoperative duration of oral alpha blockers was not significantly different between the fever and non-fever groups (56 days [range, 17–130] vs 64.5 days [range, 22–144], *p* = 0.583). Similarly, no statistically significant differences occurred in the oral dose of doxazosin. (2 mg [range, 1–14] vs 2 mg [range, 1–8], *p* = 0.246).

Among the 75 patients with Cushing syndrome, information on preoperative serum glucocorticoid and ACTH levels were available for 70, and ACTH levels were slightly lower in the non-fever group than in the fever group (4.5 pg/mL [range, 1.0–19.0] vs. 7.0 pg/mL [range, 2.7–55.0], *p* = 0.050).

Perioperative serum aldosterone levels were measured in 185 of 188 PA patients. There was no significant difference in median preoperative aldosterone levels between the non-fever and fever groups. (365 pg/mL [range, 113.0–1770.0] vs. 319 pg/mL [range, 69–1080], *p* = 0.310).

## Discussion

In the present study, we analyzed the risk factors for postoperative fever after laparoscopic adrenalectomy. Among the 406 patients examined, 124 (31%) developed postoperative fever, and only 2 (0.5%) had postoperative infections. Yi Mu et al. previously reported that the average incidence of surgical site infection was 1.9% for all surgeries [8]. The present results showed that the risk of postoperative infection in patients undergoing laparoscopic adrenalectomy was lower. In the multivariate analysis, pheochromocytoma and non-Cushing syndrome were identified as independent predictors of postoperative fever.

We attempted to examine the presence of postoperative fever by surgical technique, but only three cases of retroperitoneoscopic adrenalectomy were performed, so this was not a significant validation. The retroperitoneal approach in adrenal surgery is preferred by some urologists because of its advantages such as direct access to the adrenal gland, faster recovery, and avoidance of bowel manipulation. Li et al. analyzed 38 PA retroperitoneoscopic adrenalectomies, of which two cases (5%) had postoperative fever [9]. Further evaluation is needed in terms of surgical approach.

Only 8% of patients with Cushing syndrome developed postoperative fever in the present study. Although complex local variations in cortisol levels and the mechanisms of homeostasis in the body have not yet been fully elucidated, they are considered to have important functions in the hypothalamus and pituitary gland, such as the control of inflammation and maintenance of body temperature [10].

When the demand for steroids increases due to the resection of cortisol-producing tumors or surgical stress, acute adrenal insufficiency may occur and fever may develop [11, 12]. Furthermore, cortisol has been reported to regulate the early phase of colchicine-independent inflammation by reducing the expression of nuclear factor-kB (NF-kB), which activates inflammatory agents, such as IL-6. A physiological dose of cortisol may be necessary to prevent postoperative adrenal insufficiency and may contribute to the prevention of endogenous fever [13]. In our cohort, no significant differences were observed in preoperative serum cortisol levels between the fever and non-fever groups. Even if a tumor that produced high levels of cortisol was removed and required high dependence on exogenous cortisol postoperatively, cortisol supplementation prevented postoperative fever.

Regarding ACTH, the median preoperative plasma ACTH level was slightly lower in the non-fever group (4.5 pg/mL) than in the fever group (7.0 pg/mL) in our cohort. Patients with severe Cushing syndrome have lower ACTH levels because the compensatory rise in ACTH does not occur due to the chronic suppression of its release by the autonomous secretion of cortisol [14]. Furthermore, patients with severe Cushing syndrome are immunocompromised because high circulating levels of cortisol impair immunity [15]. In other words, patients with severe Cushing syndrome with low serum ACTH levels are immunosuppressed and less responsive to surgical invasion, which may reduce the risk of postoperative fever.

Among adrenal tumors, pheochromocytoma is empirically known to have a high incidence of perioperative fever. Smithwick et al. performed an analysis of data including patients with pheochromocytoma, and found that the incidence of perioperative fever was 73% [16]. Gordon et al. previously reported that tumor size, necrosis within tumors, high urinary MN levels, a long hospital stay prior to surgery, and non-Caucasians were valid predictors of preoperative fever after surgery for pheochromocytoma [16, 17]. In their study, they hypothesized that catecholamines increase body temperature through a combination of the induction of hypermetabolism and inhibition of heat dissipation as a result of the constriction of cutaneous blood vessels. In pheochromocytoma surgery, inflammatory cytokines, such as catecholamines and IL-6, have been implicated in the development of perioperative fever [18]. However, these studies only examined preoperative fever, not postoperative fever. Postoperative fever in patients who underwent laparoscopic adrenalectomy for pheochromocytoma has not yet been investigated. In the present study, pheochromocytoma was identified as an independent predictor of postoperative fever after laparoscopic adrenalectomy. The present results showed that more than 50% of pheochromocytoma patients had postoperative fever.

Tumor compression during surgery is associated with a high risk of intraoperative catecholamine release, which increases blood pressure. We hypothesized that preoperative hormone activity and intraoperative blood pressure correlate with postoperative fever. However, no significant differences were observed in intraoperative blood pressure or preoperative hormone activity between the fever and non-fever groups. Intraoperative blood pressure was not a risk factor for postoperative fever after pheochromocytoma surgery, but warrants further study by measuring fluctuations in intraoperative catecholamine levels.

In terms of surgical times, the univariate analysis identified the surgical time (*p* < 0.05) and pneumoperitoneum time (*p* < 0.05) as significant predictors of postoperative fever; however, the multivariate analysis did not. A prolonged surgical time for pheochromocytoma is a risk factor for catecholamine release during surgery. In the present study, we also examined surgical times in patients with pheochromocytoma in the fever and non-fever groups. However, neither the surgical nor pneumoperitoneum time were risk factors for postoperative fever, even in the univariate analysis. A prolonged surgical time has been shown to increase the risk of complications [19]. Barczyński et al. reported that decreases in surgical times by using a retroperitoneal approach resulted in shorter hospital stays and less blood loss; however, they did not examine the incidence of postoperative fever [20]. Further evaluations with a larger number of patients are needed to establish whether a prolonged surgical time is a risk factor for postoperative fever.

The present study had several limitations that need to be addressed. Since this was a retrospective study, other items that need to be analyzed, such as the type of anesthesia used, the duration of preoperative preparation medications, and intraoperative body temperature, were not available. Prospective studies are required to properly assess postoperative fever and identify optimal perioperative-related factors that avoid significant delays in diagnosis and the initiation of treatment. Furthermore, the study sample volume was small because this analysis was based on data from a single institution. Therefore, a large prospective randomized trial that focuses not only on available preoperative information, but also on intraoperative circulatory dynamics and fluctuations in catecholamine and cortisol levels is warranted.

Since postoperative fever after laparoscopic adrenalectomy is markedly affected by the hormones produced by pheochromocytoma and Cushing syndrome, it is important to carefully consider the need for treatment. Adrenal tumors are associated with a high incidence of postoperative fever; therefore, it is important to avoid the overtreatment of postoperative fever in pheochromocytoma and, conversely, undertreatment in Cushing syndrome.

## Conclusion

The present results indicate that postoperative fever after laparoscopic adrenalectomy is a common event, and infection is rarely the cause. In adrenal surgery, the presence of pheochromocytoma and Cushing syndrome may predict postoperative fever and eliminate unnecessary tests and antimicrobial administration.

## Data Availability

The datasets generated and/or analyzed during the current study are not publicly available due to medical confidentiality regarding patients’ data but are available from the corresponding author on reasonable.
